# Notch-Jagged signalling can give rise to clusters of cells exhibiting a hybrid epithelial/mesenchymal phenotype

**DOI:** 10.1098/rsif.2015.1106

**Published:** 2016-05

**Authors:** Marcelo Boareto, Mohit Kumar Jolly, Aaron Goldman, Mika Pietilä, Sendurai A. Mani, Shiladitya Sengupta, Eshel Ben-Jacob, Herbert Levine, Jose’ N. Onuchic

**Affiliations:** 1 Center for Theoretical Biological Physics, Rice University, Houston, TX 77005-1827, USA; 2 Department of Bioengineering, Rice University, Houston, TX 77005-1827, USA; 3 Department of Chemistry, Rice University, Houston, TX 77005-1827, USA; 4 Department of Physics and Astronomy, Rice University, Houston, TX 77005-1827, USA; 5 Department of Biosciences, Rice University, Houston, TX 77005-1827, USA; 6 School of Physics and Astronomy and The Sagol School of Neuroscience, Tel-Aviv University, Tel-Aviv 69978, Israel; 7 Institute of Physics, University of Sao Paulo, Sao Paulo 05508, Brazil; 8 Department of Translational Molecular Pathology, MD Anderson Cancer Center, Houston, TX 77030, USA; 9 Department of Medicine, Harvard Medical School, Boston, MA 02115, USA; 10 Harvard-MIT Division of Health Sciences and Technology, Cambridge, MA 02139, USA; 11 Division of Engineering in Medicine, Department of Medicine, Brigham and Women's Hospital, Boston, MA 02115, USA; 12 Dana Farber Cancer Institute, Boston, MA 02115, USA; 13 Metastasis Research Center, MD Anderson Cancer Center, Houston, TX 77025, USA

**Keywords:** Notch signalling, epithelial–mesenchymal transition, circulating tumour cells, hybrid epithelial/mesenchymal phenotype, multistability, cell–cell communication

## Abstract

Metastasis can involve repeated cycles of epithelial-to-mesenchymal transition (EMT) and its reverse mesenchymal-to-epithelial transition. Cells can also undergo partial transitions to attain a hybrid epithelial/mesenchymal (E/M) phenotype that allows the migration of adhering cells to form a cluster of circulating tumour cells. These clusters can be apoptosis-resistant and possess an increased metastatic propensity as compared to the cells that undergo a complete EMT (mesenchymal cells). Hence, identifying the key players that can regulate the formation and maintenance of such clusters may inform anti-metastasis strategies. Here, we devise a mechanism-based theoretical model that links cell–cell communication via Notch-Delta-Jagged signalling with the regulation of EMT. We demonstrate that while both Notch-Delta and Notch-Jagged signalling can induce EMT in a population of cells, only Jagged-dominated Notch signalling, but not Delta-dominated signalling, can lead to the formation of clusters containing hybrid E/M cells. Our results offer possible mechanistic insights into the role of Jagged in tumour progression, and offer a framework to investigate the effects of other microenvironmental signals during metastasis.

## 1. Introduction

Metastasis, the cause of 90% of cancer-related deaths [1], often begins when primary tumour cells undergo an epithelial-to-mesenchymal transition (EMT), i.e. they lose adhesion with their neighbours partially or completely and gain migratory and invasive traits, eventually entering the bloodstream as circulating tumour cells (CTCs) [2,3]. CTCs can either stay together as a cluster or migrate individually, depending on whether they have undergone a partial EMT (i.e. have residual cell–cell adhesion that enables collective cell migration as a cluster) or a complete EMT [4,5]. Upon reaching a distant organ, these CTCs exit the bloodstream and undergo a mesenchymal-to-epithelial transition (MET) that is crucial for establishing a fully grown metastasis. Such cycles of EMT and MET are a hallmark of metastatic colonization [2].

**Figure 1.**
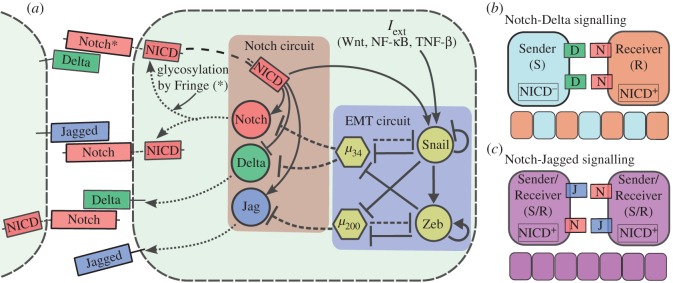
Overview of the intracellular interplay between Notch signalling pathway and EMT circuit and Notch signalling tissue patterning outcomes. (*a*) Notch signalling is activated by the interaction of the transmembrane Notch receptor with the transmembrane ligand (Delta or Jagged) of a neighbouring cell. This trans-interaction cleaves Notch and causes the release of Notch intracellular domain (NICD) into the cytoplasm. NICD then enters the nucleus where it modulates the transcription of many target genes—it activates Notch, Jagged and Snail, and inhibits Delta. Glycosylation of Notch receptor by Fringe increases the affinity of Notch to bind to Delta and reduces that to Jagged. The EMT regulatory circuit consists of two mutual-inhibitory feedback circuits, each between an EMT-inhibiting microRNA (miR) and an EMT-inducing transcription factor (TF): miR-34/SNAIL and miR-200/ZEB. Both the microRNAs translationally inhibit proteins of the Notch pathway—miR-200 inhibits Jagged, and miR-34 inhibits both Notch and Delta. EMT-inducing signals (*I*_ext_) such as Wnt and TGFβ can induce EMT by activating Snail. (*b*) Notch-Delta signalling creates an intercellular toggle switch leading neighbouring cells to adopt alternate fates—Sender cell (low Notch (receptor), high Delta (ligand)) and Receiver cell (high Notch (receptor), low Delta (ligand)), giving rise to a checkerboard-like pattern (lateral inhibition). (*c*) Notch-Jagged signalling creates an intercellular double positive feedback loop leading neighbouring cells to adopt similar fates (high Notch (receptor), high Jagged (ligand)), thereby propagating or inducing the same fate across the tissue (lateral induction).

Within individual cells, the decision as to whether cells remain epithelial, undergo partial EMT or complete EMT is mediated by various signalling pathways [6,7]. These pathways tend to converge on a core EMT regulatory network consisting of two mutually inhibitory feedback loops—one between the microRNA family miR-34 and transcription factor family SNAIL; and the other between the microRNA family miR-200 and transcription factor family ZEB (figure 1*a*). Epithelial cells have high levels of miR-34 and miR-200, and low levels of ZEB and SNAIL; mesenchymal cells have low levels of miR-34 and miR-200, and high levels of ZEB and SNAIL [8–10]. These feedback loops are interconnected—SNAIL inhibits miR-200 [8] and activates ZEB [11], while ZEB inhibits miR-34 [12]. It has been proposed that the miR-34/SNAIL loop acts as a monostable noise-buffering integrator to prevent aberrant activation of EMT, whereas the miR-200/ZEB loop acts as a tristable decision-making switch that enables three phenotypes—epithelial (no EMT: high miR-200, low ZEB), mesenchymal (complete EMT: low miR-200, high ZEB) and hybrid epithelial/mesenchymal (E/M) (partial EMT: medium miR-200, medium ZEB) [13].

Importantly, the regulation of EMT/MET is influenced by many non-cell-autonomous factors such as extracellular matrix density and stiffness, stromal factors and cell–cell communication [14–16]. Among those various pathways, Notch signalling serves as a key regulator and mediates cell–cell communication both between cancer cells themselves, and between the tumour and stroma [15,17]. The Notch pathway gets activated when the receptor of one cell—Notch—interacts with the ligand of another cell—Delta or Jagged, leading to the cleavage of Notch and consequent releases of Notch intracellular domain (NICD). NICD then enters the nucleus and regulates the expression of many Notch target genes [18], including Delta and Jagged; it represses Delta [19] but activates Jagged [20]. Consequently, Notch-Delta (N-D) signalling gives rise to a double negative feedback loop between the two cells and drives them to adopt different fates—one cell becomes a Sender (high ligand (Delta), low receptor (Notch)) and the other a Receiver (low ligand (Delta), high receptor (Notch)). Conversely, Notch-Jagged (N-J) signalling forms a double positive feedback loop between the two cells and drives them to adopt a similar fate—hybrid Sender/Receiver (high ligand (Jagged), high receptor (Notch)) that allows neighbouring cells to both send and receive signals [21,22].

The Notch and EMT circuits are highly interconnected—NICD activates SNAIL [23,24], miR-200 inhibits Jagged [25] and miR-34 inhibits both Notch and Delta [26,27], thereby indicating how the regulation of EMT/MET can be highly dependent on cell–cell communication via Notch signalling. However, most experimental and theoretical studies for EMT have focused only on cell-autonomous decisions [8–10,13,28–33]; therefore, how cell–cell communication might affect EMT/MET regulation and consequently the spatial organization of E, E/M and M cells remain elusive.

Here, we devise a theoretical framework that couples Notch-Delta-Jagged (N-D-J) signalling with the EMT/MET regulation. We show that the epithelial cells usually behave as Senders (S) or Receivers (R) only, but not as hybrid Sender/Receivers (S/R). Activation of Notch signalling by either ligand—Delta or Jagged—can induce a cell to undergo a partial or complete EMT and these cells in a partial EMT (i.e. hybrid E/M cells) or a complete EMT (i.e. mesenchymal cells) usually behave as hybrid S/R, i.e. they can both send as well as receive signals via Notch signalling. Finally, our simulations demonstrate that Jagged-dominated signalling but not Delta-dominated signalling can induce as well as maintain a cluster of cells in the hybrid E/M phenotype, hence pointing out the possible role of Jagged in formation and maintenance of CTC clusters.

## 2. Results

### 2.1. Epithelial-to-mesenchymal transition-inducing signals can activate Notch signalling

**Figure 2.**
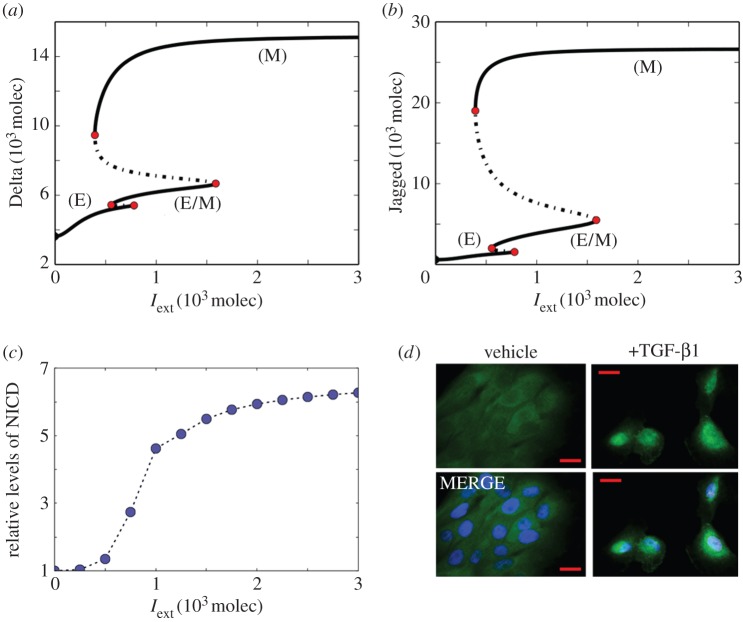
Activation of Notch pathway via EMT inducer signal (*I*_ext_). Bifurcation curves of the levels of (*a*) Delta and (*b*) Jagged as a function of EMT inducer levels (*I*_ext_), for a one-cell system in the absence of external ligands (*D*_ext_ = *J*_ext_ = 0, *N*_ext_ = 5000). Increasing *I*_ext_ induces a partial or complete EMT and concomitant increase in levels of Jagged and Delta. The EMT phenotypes are defined based on the levels of miR200, miR34, Snail and Zeb, presented in electronic supplementary material, figure S1. (*c*) Relative average levels of NICD (*I*) for a simulated two-dimensional layer of 50 × 50 cells for different levels of *I*_ext_. The cells were simulated in a hexagonal lattice, starting from random initial conditions and the levels of NICD were measured after 120 h. The values of all parameters are presented in electronic supplementary material, table S1. (*d*) Immunofluorescence images of NICD (green) and cell nuclei (blue) for MCF10A cells treated with 5 ng ml^−1^ TGF-β1 for 6 days.

As a first step towards elucidating the interplay between Notch signalling and the core EMT circuit, we evaluate how EMT-inducing signals such as Wnt and TGFβ affect the levels of the ligands Jagged and Delta. We initially simulated the case of an individual cell that is exposed to an EMT-inducing signal (*I*_ext_); this cell is being treated in isolation, i.e. no coupling to Notch ligands from the neighbouring cells. High levels of *I*_ext_ decrease the EMT-inhibiting microRNAs miR-34 and miR-200 (electronic supplementary material, figure S1) and consequently induce a partial or complete EMT (hybrid E/M or M phenotype, respectively). Decreased levels of microRNAs relieve the repression on Delta and Jagged, leading to an increase in both Delta and Jagged (figure 2*a*,*b*). Thus, induction of EMT in a given cell increases the levels of Notch ligands that can activate Notch signalling in the adjacent cells.

Next, we simulated a bidimensional layer of 2500 (=50 × 50) cells that interact among each other via Notch-Delta-Jagged signalling, and measured the levels of active Notch signalling (NICD) for different values of *I*_ext_. Our simulations show that increased levels of the driving signal *I*_ext_ lead to increased levels in average of Notch signal (NICD) (figure 2*c*). To validate this prediction experimentally, we treated human breast epithelial MCF10A cells with TGFβ1, a well-known EMT inducer. The treated cells expressed higher levels of NICD as compared to the control (figure 2*d*), indicating that inducing EMT can activate Notch signalling in a population of cells.

### 2.2. Notch-Delta and Notch-Jagged signalling induces epithelial-to-mesenchymal transition

**Figure 3.**
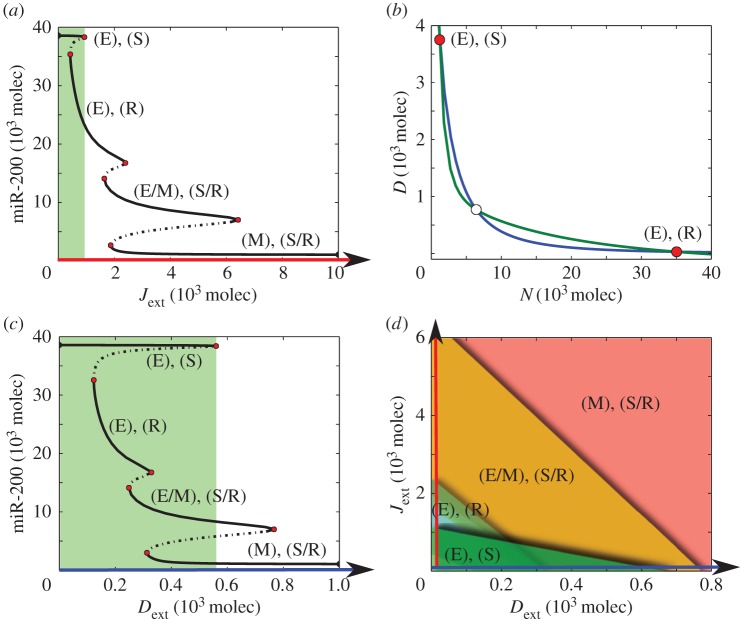
Bifurcation curves, nullcline and phase diagram. (*a*) Bifurcation curve of the levels of miR-200 as a function of the number of external Jagged (*J*_ext_) for *D*_ext_ = 0 and *N*_ext_ = 5000 molecules. At low *J*_ext_, the cell adopts the epithelial (E) phenotype where it can be either a Sender (S) (high Delta, low Notch) or Receiver (R) (low Delta, high Notch)—(E),(S) or (E),(R). At increased levels of *J*_ext_, the cell undergoes a transition to the hybrid epithelial/mesenchymal (E/M) phenotype. In this state, the cell presents high levels of both Notch and Jagged (see electronic supplementary material, figure S2), therefore adopting a hybrid Sender/Receiver (S/R) state—(E/M), (S/R). Further increase in the levels of *J*_ext_ induces a complete EMT and the cells adopt the mesenchymal (M) phenotype and also the S/R state—(M), (S/R). (*b*) Nullclines for the case of low levels of *J*_ext_ (*J*_ext_ = 600, *D*_ext_ = 0, *N*_ext_ = 5000 molecules). The cell is in an epithelial phenotype, and can be either a Sender (high Delta, low Notch) or Receiver (low Delta, high Notch). Blue nullcline is for the condition of all ODEs being set to zero except for d*D*/d*t* and green nullcline is for the condition of all ODEs being set to zero except for d*N*/d*t*. Unfilled circles represent unstable steady states, whereas red filled circles represent the two stable states: Sender (high Delta, low Notch) and Receiver (low Delta, high Notch). (*c*) Bifurcation curve of the levels of miR-200 as a function of the number of external Delta (*D*_ext_) for *J*_ext_ = 0 and *N*_ext_ = 5000 molecules. Green rectangle represents the range of parameter for the existence of Epithelial-Sender (E-S) phenotype. (*d*) Two-parameter bifurcation diagram (phase diagram) as a function of external Delta (*D*_ext_) and external Jagged (*J*_ext_). Each colour represents a different state: (E),(S) (dark green), (E),(R) (light green), (E/M),(S/R) (yellow) and (M),(S/R) (red).

Next, to discern how activating Notch signalling affects the core EMT circuit in a single cell, we evaluate the dynamics of the coupled circuit as a function of fixed levels of external ligands—*D*_ext_ and *J*_ext_—representing the concentration of Delta and Jagged, respectively, on neighbouring cells. An increase in *J*_ext_ can enhance the levels of NICD and lead to a partial EMT and eventually a complete EMT by increasing SNAIL (figure 3*a*). Interestingly, for low levels of *J*_ext_, cells in the epithelial phenotype (E) can attain one of the two equilibrium states—(i) (high Delta, low Notch) and (ii) (low Delta, high Notch), i.e. the cell can act either as a Sender (S) or as a Receiver (R) of Notch signalling (figure 3*a*,*b*). However, when the cell undergoes a partial or complete EMT, it has (high Notch, high Jagged) and can act both as a Sender as well as Receiver of the Notch signalling, i.e. it adopts a hybrid Sender/Receiver (S/R) phenotype (electronic supplementary material, figure S2*a*,*b*). Because cells in the hybrid S/R state can induce the same fate as theirs in their neighbouring cells through lateral induction [21,34], we hypothesize that Notch-Jagged, but not Notch-Delta signalling is likely to form clusters of partial EMT (hybrid E/M) cells or complete EMT (M) cells.

Similarly, increasing *D*_ext_ instead of *J*_ext_ also leads to a partial or complete EMT in the cell and it adopts the hybrid Sender/Receiver (S/R) state. However, signalling though Delta (*D*_ext_) expands the range of parameters for the existence of an Epithelial-Sender (E-S) state (compare the width of the green rectangle in figure 3*c* versus that in figure 3*a*; also see figure 3*d*). This large region of coexistence between states with high Delta (E-S) levels and with low Delta levels (E-R, E/M-S/R, M-S/R) is typical of Notch-Delta interactions [21,22].

### 2.3. Jagged-dominated Notch signalling can give rise to clusters of hybrid epithelial/mesenchymal cells

**Figure 4.**
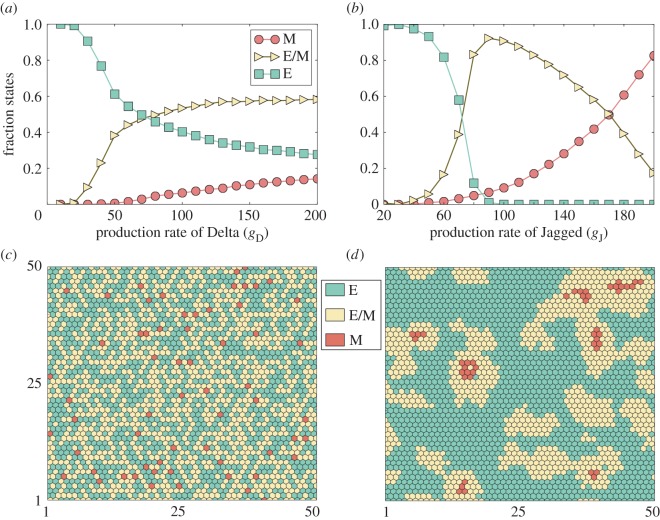
Tissue patterning for Delta-dominated and Jagged-dominated Notch signalling. Simulation of a two-dimensional layer of 50 × 50 cells interacting via Notch signalling. Fraction of cells for each phenotype: epithelial (E), epithelial/mesenchymal (E/M) and mesenchymal (M) for different production rates of (*a*) Delta and (*b*) Jagged. (*c*) Snapshot of the simulated tissue representing the spatial distribution of E, E/M and M cells for *g*_D_ = 70 and *g*_J_ = 20 molecules h^−1^. (*d*) Same as (*c*) for *g*_D_ = 20 and *g*_J_ = 70 molecules h^−1^. The initial levels of the proteins for each cell are initially chosen randomly (electronic supplementary material, figure S3) and the values are measured after an equilibrium time of 120 h. In the absence of any other external signal, the clusters of E/M are transient and disappear after 240 h (electronic supplementary material, figure S4).

To better characterize the different possible roles of inducing EMT via Notch-Delta versus Notch-Jagged signalling, we evaluate the dynamics of the Notch-EMT coupled circuit at the tissue level by simulating a two-dimensional layer of epithelial cells interacting via Notch signalling. The initial configuration of each cell was chosen randomly and the same initial condition was used for all simulations (electronic supplementary material, figure S3). These simulations were done at many different levels of production rates for Delta and Jagged in order to mimic situations of Delta-dominated and Jagged-dominated signalling prevalent in the population. At low production levels of both Delta and Jagged, all cells retain their epithelial phenotype after 120 h (figure 4*a*,*b*). Increasing the production levels of either of the ligands activates Notch signalling and consequently increases the number of cells that undergo a partial or complete EMT, i.e. number of cells in the E/M and M phenotypes (figure 4*a*,*b*). On investigating the spatial distribution of the E, E/M and M phenotypes in the two-dimensional layer, we observe that when Notch-Jagged signalling dominates, most cells in the hybrid E/M or M phenotype tend to form clusters among themselves; but when Notch-Delta signalling dominates, such cells are spatially segregated and few, if any, clusters are observed (figure 4*c*,*d*). These results suggest that the cells that undergo partial or complete EMT tend to aggregate forming clusters when Jagged-driven Notch signalling dominates over the Delta-driven one. However, in the absence of any external EMT inducer, those clusters are transient and the cells tend to lose their E/M or M phenotype and eventually become epithelial (electronic supplementary material, figure S4). As we show in the following sections, an external signal that either induces EMT or activates Notch signalling can stabilize these clusters of cells.

**Figure 5.**
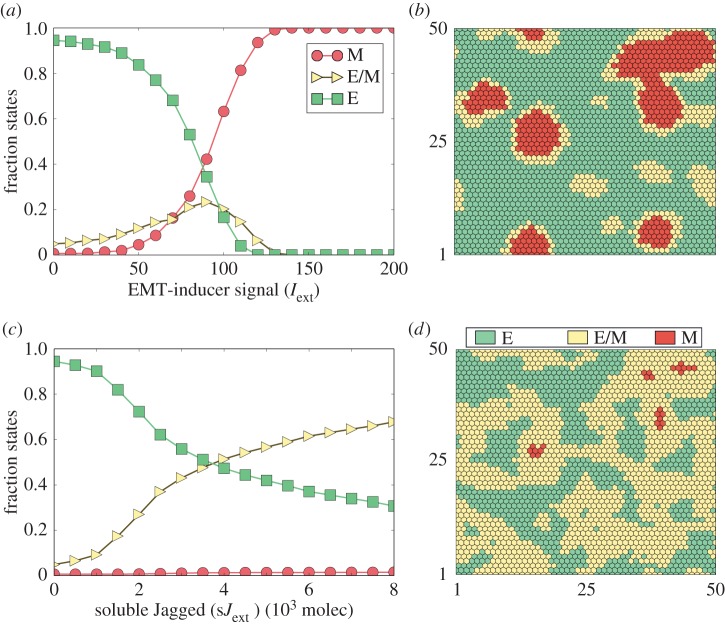
Effect of external inducers of the Notch-EMT coupled circuit on tissue patterning. Simulation of a two-dimensional layer of 50 × 50 cells interacting via Notch-Delta-Jagged signalling. (*a*) Fraction of cells for each phenotype: epithelial (E), epithelial/mesenchymal (E/M) and mesenchymal (M) for different levels of an EMT-inducer signal (*I*_ext_). (*b*) Snapshot of the simulated tissue representing the spatial distribution of E, E/M and M cells for *I*_ext_ = 70 molecules, *g*_D_ = 20 and *g*_J_ = 70 molecules h^−1^. A majority of the cells that undergo EMT adopt the M state. (*c*) Fraction of cells for each phenotype for different levels of external soluble Jagged (s*J*_ext_) for *I*_ext_ = 0 molecules, *g*_D_ = 20 and *g*_J_ = 70 molecules h^−1^. (*d*) Snapshot of the simulated tissue representing the spatial distribution of E, E/M and M cells s*J*_ext_ = 4000 molecules. The levels were measured after 120 h, starting from the configuration presented in figure 4*c*.

We further evaluate the stability of these clusters (presented in figure 4*d*) in the presence of two types of external signal: (i) an external EMT inducer (*I*_ext_) that activates Snail and (ii) soluble ligands (Delta and Jagged) that bind to the Notch receptor and activate Notch signalling. Applying *I*_ext_ increases the number of cells undergoing a partial and complete EMT, irrespective of whether the intercellular signalling is dominated by Delta or Jagged (figure 5*a*; electronic supplementary material, S5A). Consistently, Jagged-dominated signalling predominantly leads to the clusters of non-epithelial cells; while Delta-dominated signalling results in ‘salt-and-pepper’ patterns of epithelial and mesenchymal cells (figure 5*b*; electronic supplementary material, S5B).

Notch signalling can also be activated in a paracrine way, i.e. via soluble ligands secreted by other cells [35]. Hence, we further evaluate the effect of paracrine activation of Notch on EMT induction and spatial patterns observed in the layer of cells. Higher levels of soluble Jagged leads to an increase in the population of hybrid E/M cells, but not mesenchymal cells (figure 5*c*), unlike the case when EMT is induced via activation of SNAIL by *I*_ext_ (figure 5*a*). Consequently, the clusters observed are mostly composed of hybrid E/M cells (figure 5*d*). Similar behaviour is observed in the presence of soluble Delta (electronic supplementary material, figure S6); again, clusters are more prominently observed in Jagged-dominated signalling (electronic supplementary material, figures S5C,D and S6).

Notch-Delta signalling and Notch-Jagged signalling canonically have different signalling feedbacks thereby leading to different patterns—lateral inhibition and lateral induction, respectively. However, we found that both soluble Delta and Jagged similarly affect the formation of cell clusters. These differences can be attributed to the different dynamics of juxtacrine versus paracrine signalling between Notch and its ligands. When the soluble ligands (both Delta and Jagged) bind to Notch receptor in a distant cell, they cause the release of NICD, and consequently activate SNAIL, Jagged and Notch, but repress Delta in that ‘target’ cell. Therefore, the ‘target’ cells—irrespective of whether they have been activated by soluble Jagged or soluble Delta—are likely to have (high Notch, high Jagged, low Delta) levels, a signature commensurate with the cells in a hybrid E/M phenotype. Consequently, ‘target’ cells of soluble ligands participate predominantly in Notch-Jagged signalling.

Overall, Jagged-dominated Notch signalling enables cluster formation of hybrid E/M cells, an effect that is mitigated by Fringe, a glycosyltransferase that increases the binding affinity of Notch for Delta, but decreases that for Jagged (electronic supplementary material, figure S7).

**Figure 6.**
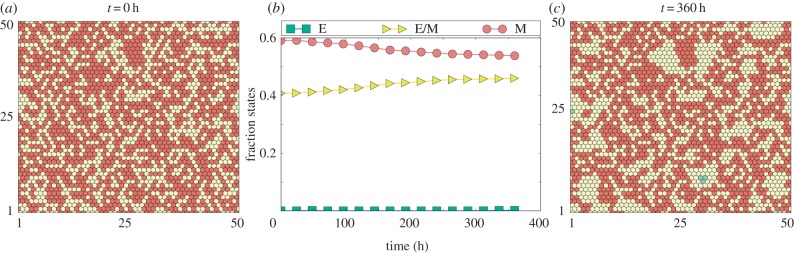
Notch-Jagged signalling acts as a ‘phenotypic stability factor’ for the hybrid E/M phenotype. Simulation of 50 × 50 cells interacting via N-D-J signalling. (*b*) Fraction of cells adopting epithelial (E), epithelial/mesenchymal (E/M) and mesenchymal (M) phenotypes at different time points for the given initial condition. (*a*,*c*) Levels of miR200 for 50 × 50 hexagonal lattice at *t* = 0 and *t* = 360 h. Red cells are in an M phenotype, yellow ones in a hybrid E/M one.

Next, we investigated how Delta-dominated and Jagged-dominated signalling affect the spatial patterning when most cells are in a partial or complete EMT phenotype to begin with. In the case of Delta-dominated signalling, many cells undergo MET to adopt an epithelial phenotype, and the epithelial and non-epithelial cells arrange largely into a ‘salt-and-pepper’ pattern (electronic supplementary material, figure S8). By contrast, for Jagged-dominated signalling, MET rarely happens; rather the initial random distribution patterns of E/M and M self-organize to form clusters of E/M cells (figure 6*a*; electronic supplementary material, S9). These clusters can then be stabilized by Notch-Jagged signalling via lateral induction; therefore, Notch-Jagged signalling can not only induce but also maintain the cluster of hybrid E/M cells; or Notch-Jagged signalling can potentially act as a ‘phenotypic stability factor’ [36] for the hybrid E/M phenotype.

### 2.4. Implications of Jagged-dominated Notch signalling as a ‘phenotypic stability factor’

**Figure 7.**
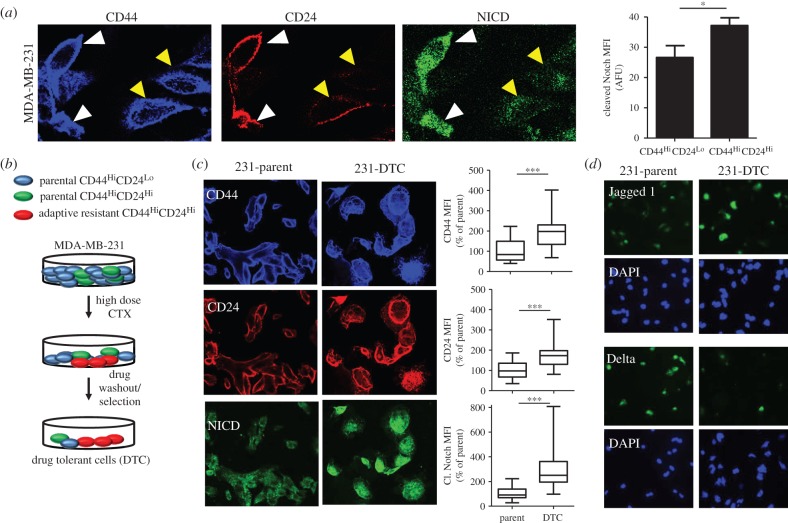
(*a*) Representative confocal microscopy shows CD44, CD24 and cleaved notch (NICD) in a population of drug naive MDA-MB-231. Yellow arrows indicate CD44^Hi^CD24^Lo^ (M) population of cells and the white arrows indicate the CD44^Hi^CD24^Hi^ (E/M) cells. Histogram (right panel) shows quantification of NICD in the distinct phenotype populations (M versus E/M). *N* = 3 biological replicates. (*b*) Schematic describes the experimental protocol to generate drug-tolerant cells (DTCs) parental MDA-MB-231 cells were treated with docetaxel at 100 nM (20× the IC50) and subsequently selected by substrate re-attachment and acute population outgrowth. (*c*) Representative confocal microscopy shows CD44, CD24 and NICD in the MDA-MB-231 parent and DTC populations. Right panel shows quantification of fluorescence intensity of each signal determined by at least 25 individual fields. *N* = 3 biological replicates. (*d*) Representative confocal microscopy shows Jagged and Delta expression in MDA-MB-231 parent and DTC. DAPI nuclear stain (blue). *N* = 3 biological replicates.

Previously, we demonstrated that ‘phenotypic stability factors’ maintain the ‘metastable’ hybrid E/M phenotype [33] which can also associate to higher tumour-initiating ability (also known as stemness) [37,38]. Cells co-expressing CD24 (epithelial marker) and CD44 (mesenchymal marker), CD24hi CD44hi, have been shown to correspond to a hybrid E/M phenotype [39] and possess higher tumour-initiation potential *in vitro* [39] and *in vivo* [40]. Here, we investigated the levels of Notch signalling in two distinct cell lines with different phenotypic basal states. Primarily, we determined that the mesenchymal-like breast cancer cell line, MDA-MB-231, which display a predominant CD44^Hi^CD24^Lo^ phenotype, differentially express higher NICD levels in the E/M phenotype than the M phenotype (figure 7*a*). To support these evidences, we analysed the epithelial-like MDA-MB-468 cells, which are predominantly CD44^Hi^CD24^Hi^, and determined that Jagged expression was clustered, confirming the association between these phenotypic states, as analysed by confocal microscopy (electronic supplementary material, figure S10).

The E/M, tumour-initiating phenotype has also been shown to be associated with drug resistance [41]. To test the role for Jagged-dominated Notch signalling in drug resistance, experimentally, we used an *in vitro* model in which cancer cells have an induced drug-tolerant hybrid E/M phenotype that displays high tumour-initiating capability [40]. As shown in figure 7*b* schematic, MDA-MB-231 cells were exposed to a high dose of docetaxel—a cytotoxic chemotherapy used in the first-line treatment of triple negative breast cancer (TNBC)—followed by substrate reattachment and acute population outgrowth, which results in a population of drug-tolerant cells (DTCs) [40] (figure 7*b*).

Consistent with earlier reports [40], we confirmed that DTCs have higher expression of CD24 (epithelial marker) and CD44 (mesenchymal marker) as compared to the parent population, indicating a shift towards the hybrid E/M phenotype, as determined by confocal microscopy (figure 7*c*). Interestingly, we observed that DTCs expressed higher NICD and Jagged, but less Delta, as compared to the parent population (figure 7*c*,*d*). These data support the hypothesis that Jagged-dominated Notch signalling may be crucial to maintain the hybrid E/M phenotype and also associates cells with a higher likelihood of gaining stemness, as defined by the traits of heightened drug resistance as well as tumour initiation.

## 3. Discussion

Notch signalling is an evolutionarily conserved cell–cell communication pathway that is involved in multiple hallmarks of cancer. Recent studies have highlighted that the two ligand families—Delta and Jagged—can play different and sometimes opposing roles in mediating cell-fate determination via Notch signalling [42]. Ours, to the best of our knowledge, is the first study that elucidates the different roles of the ligands Delta and Jagged in epithelial plasticity (EMT/MET), a hallmark of cancer metastasis.

Our results suggest that Notch signalling can induce EMT via both Delta and Jagged, but inducing EMT through Jagged can specifically enable the formation of clusters of cells in a hybrid E/M phenotype. The formation of these clusters is enhanced and their stability is prolonged by EMT-inducing signals and/or soluble ligands of Notch signalling pathway. Notch-Jagged signalling is usually involved in lateral induction [20,34,43,44], i.e. inducing the neighbour to adopt the same cell fate as that of its own. Thus, a cluster of cells with Jagged-dominated Notch signalling can mutually stabilize their cell fate. Such a mutual stabilization among the cells in a ‘metastable’ partial EMT or hybrid E/M phenotype can lead to formation of clusters of CTCs and is hence of critical clinical relevance.

The CTCs displaying a hybrid E/M phenotype have been found in the bloodstream of lung, breast and prostate cancer patients [5,45–47], and they can lead to clusters of CTCs due to their ability to undergo collective migration. Such clusters are apoptosis-resistant, can exit the bloodstream relatively easily, can be up to 50 times more metastatic than individually migrating CTCs (in mesenchymal phenotype), and, therefore, pose a much higher metastatic risk in patients [5,48,49]. With an increasing appreciation of the notion that EMT is not an ‘all-or-none’ response and that cancer cells *in vivo* rarely undergo complete EMT [7,50,51], cancer cells might as well prefer to stay in a hybrid E/M phenotype owing to the above-mentioned advantages. Therefore, maintaining the cells in a hybrid E/M phenotype, otherwise considered to be ‘metastable’ [52], can offer many key survival advantages to a cluster of CTCs. We predict that these advantages can be potentially mitigated by therapeutic targeting of Jagged1.

Therapeutic targeting of Jagged1 is not only expected to possibly ‘break’ these clusters to solitarily migrating CTCs (mesenchymal phenotype), but also subdue their tumour-initiating potential. Recent studies show that the cells in a hybrid E/M phenotype (identified by CD24^+^/CD44^+^) can form much more tumours than those in a purely mesenchymal phenotype (identified by CD24^−^/CD44^+^), especially when the hybrid E/M phenotype is stabilized, for instance, by ‘phenotypic stability factor’ [36] such as OVOL [33,37–40]. Our experimental data showing that the drug-tolerant population of MDA-MB-231 is CD24^+^/CD44^+^ and has elevated levels of Jagged1 and Notch suggest that Notch-Jagged signalling also acts as an intercellular ‘phenotypic stability factor’ for the hybrid E/M phenotype; and is resonant with the emerging notion that carcinoma cancer stem cells (CSCs) lie mid-way on the ‘EMT axis’ [7,37,53–55], and that Notch-Jagged signalling is often implicated in maintaining CSC population and chemoresistance [15,35].

Furthermore, targeting Jagged1 can also mollify the effects of many tumour-promoting inflammatory cytokines that increase Notch-Jagged signalling by activating Jagged and/or inhibiting Delta [42,56,57]. Hence, Jagged1 can be a critical therapeutic target to halt aggressive tumour progression [58], and targeting Jagged1 specifically, as recently attempted [59], can mitigate the side effects of targeting the entire Notch pathway by inhibiting NICD [60]. However, Notch-Jagged (N-J) signalling is not specific to pathological situations such as cancer metastasis. For instance, N-J signalling can be crucial in spatial patterning during the development of inner ear [34], pancreas [61] and epidermal stem cell clusters [62]. Thus, the results presented here might also be applicable to elucidate the role of Jagged during epithelial organization and homeostasis in multiple biological contexts.

We note that the major goal of this work is the formulation of a new theoretical framework that allows us to consider the role of Notch signalling in spatially coordinating the EMT response. We have used limited experimental data to qualitatively validate some of our underlying assumptions related to the different roles of Delta and Jagged and to the ability of NICD to drive EMT. Future experimental work will provide more quantitative tests of our emerging picture, in particular with regard to the predicted spatial correlation. Also, a causal role of Notch-Jagged signalling in mediating tumour-initiation potential and/ or drug resistance of the CD24^+^ CD44^+^ hybrid E/M cells remains to be directly tested.

To conclude, we show that Notch-Jagged signalling can induce and maintain a cluster of cells in a partial EMT phenotype, thereby suggesting the potential role of Jagged1 in stabilizing the clusters of CTCs, the primary ‘bad agents’ of metastasis [5,7]. To the best of our knowledge, ours is the first theoretical study elucidating how the intracellular regulation of EMT is affected by any form of intercellular communication. Our theoretical framework proposes a critical therapeutic target and can be further used to investigate the effect of external factors such as inflammation on the formation of such clusters [4], as well as to predict likely spatial positions of different types of CSCs in the tumour mass [63]. Finally, our cell–cell communication framework can be integrated with the population-level mathematical models of CSCs [64–67] to elucidate the collective or cooperative behaviour in cancer cell colonies [68,69].

## 4. Material and methods

### 4.1. Theoretical framework

The equations for the mathematical model are presented in electronic supplementary material, section S1. The values of the parameters used for the model are given in electronic supplementary material, section S2. The computational analysis was performed in Python and the source codes are freely available on Github (https://github.com/mboareto/Notch-EMT). Bifurcations for the one-cell system were evaluated using PyDSTool [70].

### 4.2. Cell culture

MCF10A cells were maintained in DMEM/F12 media (Sigma-Aldrich) supplemented with 5% horse serum, 20 ng ml^−1^ epithelial growth factor, 0.5 mg ml^−1^ hydrocortisone, 100 ng ml^−1^ cholera toxin, 10 µg ml^−1^ insulin and penicillin/streptomycin (1%). To induce EMT, they were treated with vehicle or 5 ng ml^−1^ of TGF-β1 (R&D systems) for 6 days.

MDA-MB-231 and MDA-MB-468 cells (ATCC) were cultured in DMEM containing 10% fetal bovine serum at 37°C and 5% CO_2_. During treatments with chemotherapeutics, cells were grown to semi-confluence and treated with indicated concentrations of chemotherapy in serum-containing medium for indicated time points. For generation of DTCs, cells were treated for 48 h with docetaxel (100 nM). Following washes with phosphate-buffered saline (PBS), adherent cells were trypsinized and re-plated at a density of 1.5–2 × 10^5^ cells ml^−1^ and cultured in serum-containing medium onto glass slides (BD, San Jose, CA, USA). After 24 h incubation, floating cells were removed and remaining cells were washed with 1× PBS and considered as chemotherapy-tolerant cells. Populations of drug naive parent cells were always cultured alongside DTC and fresh media was added at every interval that the experimental population (DTC) received fresh media.

Unless noted otherwise, all reagents and chemotherapies were of the highest grade purchased from Sigma-Aldrich (St Louis, MO, USA). All chemotherapeutics were dissolved in dimethylsulfoxide to a stock concentration of 10 mM and kept frozen before fresh preparation into working concentration in DMEM.

### 4.3. Confocal microscopy and immunofluorescence

Parent cells or DTCs were generated as described above and plated in four chamber glass slides (BD Biosciences, San Jose, CA, USA) at a concentration of 10 000 cells ml^−1^. Following treatments, cells were washed in PBS and fixed in 4% paraformaldehyde for 30 min. Permeabilization, when necessary, was achieved with 10% (v/v) goat serum (Vector Laboratories, Burlingame, CA, USA) and 0.05% Saponin (w/v) in PBS for 90 min. Blocking was performed in 10% (v/v) goat serum in PBS. The cells were labelled with the indicated fluorescently conjugated primary antibodies CD44 (Clone IM7 from eBioScience) at 1 : 500, CD24 (clone ML5 from eBioScience) at 1 : 100, Jagged-1 (cat# 200-401-698S from Rockland, Limerick, PA, USA), Delta at 1 : 100 (clone H-265 from Santa Cruz Biotech, Dallas, TX, USA), cleaved notch at 1 : 50 (clone ab8925 from Abcam, Cambridge, MA, USA) for 24 h at 4°C and masked with hard-set mounting medium (Vector Laboratories, Burlingame CA). Bright field and fluorescent images were obtained using three channels on a Nikon Eclipse TI-U microscope with a 20× ELDW, 10× or 40× Plan-Apo objective lens (Nikon, Melville, NY, USA). NIS Elements Viewer version 3.22 (Nikon) software was used to capture the images to file. Confocal microscopy of IHC from frozen sections of tumour tissue was performed with an inverted Nikon confocal microscope (TE2000) with Auto DeBlur deconvolution software and fitted with three laser detection (Nikon). Gains were set manually based on negative control stains (secondary antibody only) and were left unaltered between treatment groups of similar experiments. When representative images are shown in figures, these are derived from experiments performed in at least biological triplicate on independent occasions. Quantification of the fluorescent intensity was determined using Adobe CS5 software (San Jose, CA, USA) and confirmed using ImageJ software (NIH) and indication of CD44 Hi/Lo or CD24Hi/Lo was determined by relative fluorescent intensities between individual cells.

For immunofluorescence staining of MCF10A cells, they were plated on pre-sterilized coverslips and were fixed and permeabilized with 4% paraformaldehyde + 0.1% Triton-X 100 for 10 min at room temperature (RT). PFA was quenched by 5% glycine 15 min RT and samples were blocked with 4% bovine serum albumin (BSA) in PBS for 1 h RT. Primary antibody against cleaved Notch-1 (NICD, Cell Signaling Technology) was diluted 1:1000 in 4% BSA in PBS and incubated overnight at 4°C. Species-specific Alexa Fluor 488—conjugated secondary antibody (Life Technologies) was diluted 1 : 1000 in 4% BSA in PBS and incubated 1 h RT. Nuclei were counterstained with 4′,6-diamidino-2-phenylindole (DAPI; Molecular Probes). The coverslips were mounted onto glass slides with DAKO fluorescent mounting medium (DAKO).

### 4.4. Statistics

Statistical analysis was performed using Prism software (Graphpad, La Jolla, CA, USA) determined by ANOVA followed by a Newman–Keuls *post hoc* test when values were represented between multiple groups and Student's *t*-test used to identify statistical significance between individual groups. The data are expressed as a mean ± s.e.m.

## Supplementary Material

Supplementary Information
