# Supplementary material for: Notch-Jagged signalling can give rise to clusters of cells exhibiting a hybrid epithelial/mesenchymal phenotype
Source: J R Soc Interface. 2016 May;13(118):20151106. doi: 10.1098/rsif.2015.1106 (PMC4892257; doi:10.1098/rsif.2015.1106)
Supplement: Supplementary Information [file rsif20151106supp1.pdf]

Supplementary Information

S1 - The Theoretical framework

Here, we integrated our previously introduced Notch-Delta-Jagged circuit [1, 2] with the EMT core regulatory circuit [3], in order to evaluate the role of cell-cell communication on EMT. In this section, we describe the equations for the integrated circuit. In section S2, we present the values of each parameter. In section S3, we detailed how both circuits are integrated.

The equations that describe the amount of Notch (N), Delta (D), Jagged (J), NICD (I) are given by:

$$\frac{dN}{dt} = k_P g_N H^S(I,N) P_l(\mu_{34},2) - N[(k_{cD}D + k_{cJ}J) + (k_{tD}D_{ext} + k_{tJ}J_{ext})] - \gamma N \tag{S1}$$

$$\frac{dD}{dt} = k_P g_D H^S(I,D) P_l(\mu_{34},3) - D(k_{cD}N + k_{tD}N_{ext}) - \gamma D \tag{S2}$$

$$\frac{dJ}{dt} = k_P g_J H^S(I,J) P_l(\mu_{200},5) - J(k_{cJ}N + k_{tJ}N_{ext}) - \gamma J \tag{S3}$$

$$\frac{dI}{dt} = N(k_{tD}D_{ext} + k_{tJ}J_{ext}) - \gamma_I I \tag{S4}$$

were γ represents the degradation rate of Notch, Jagged and Delta, and γ_I the degradation of the signal (NICD). k_{cD} and k_{cJ} are the *cis*-inhibition rate for Notch-Delta and Notch-Jagged interactions respectively, and k_{tD} and k_{tJ} are the *trans*-activation rate for Notch-Delta and Notch-Jagged interactions respectively and are functions of the signal (NICD) given by: $k_t(I) = k_t H^S(I, \lambda^F)$ where λ^F represents the effect of Fringe (for details see [1]). k_P represents the translation rate; and g_N, g_D, and g_J are the transcription rates of Notch, Delta and Jagged respectively. N_{ext}, D_{ext} and J_{ext} represent the amount of protein available for binding - which can be on the membrane surface of neighboring cells or in a soluble form. We considered that the modulation of the production rates are mediated by a shifted Hill function defined as $H^S(A,B) = H^-(A) + \lambda_{A,B}H^+(A)$ [3]. This function represents the modulation of A on B where the weight factor $\lambda_{A,B}$ represents the fold-change in production rate of B due to the effect of A. Therefore, for activation, $\lambda > 1$; for repression, $\lambda < 1$; and for no effect, $\lambda = 1$. The functions $H^+(x) = \frac{(x/x_0)^n}{1+(x/x_0)^n}$ and $H^-(x) = \frac{1}{1+(x/x_0)^n}$ are respectively positive and negative Hill functions. $P_l(\mu, n)$ represent the translation inhibition due to the binding of miRNA with n binding sites (for details see Supplementary Information of [3]).

The equations for the EMT circuit, that represents the amount of miR200 (μ_{200}), miR34 (μ_{34}), Snail (S) and Zeb (Z) are given by:

$$\begin{aligned}\frac{d\mu_{200}}{dt} =& g_{\mu_{200}} H^S(Z,\mu_{200}) H^S(S,\mu_{200}) - g_Z H^S(Z,Z) H^S(S,Z) P_y(\mu_{200},6) \\ &- g_J H^S(I,J) P_y(\mu_{200},5) - \gamma_{\mu_{200}}\mu_{200}\end{aligned} \tag{S5}$$

$$\begin{aligned}\frac{d\mu_{34}}{dt} =& g_{\mu_{34}} H^S(S,\mu_{34}) H^S(Z,\mu_{34}) - g_S H^S(S,S) H^S(I,S) H^S(I_{ext},S) P_y(\mu_{34},2) \\ &- g_D H^S(I,D) P_y(\mu_{34},3) - g_N H^S(I,N) P_y(\mu_{34},2) - \gamma_{\mu_{34}}\mu_{34}\end{aligned} \tag{S6}$$

$$\frac{dZ}{dt} = k_P g_Z H^S(Z,Z) H^S(S,Z) P_l(\mu_{200},6) - \gamma_Z Z \tag{S7}$$

$$\frac{dS}{dt} = k_P g_S H^S(S,S) H^S(I,S) H^S(I_{ext},S) P_l(\mu_{34},2) - \gamma_S S \tag{S8}$$

where $g_{\mu_{200}}$, $g_{\mu_{34}}$, g_Z and g_S represents the production rate of miR200, miR34, Zeb and Snail, respectively; $\gamma_{\mu_{200}}$, $\gamma_{\mu_{34}}$, γ_Z and γ_S represents the degradation rate of miR200, miR34, Zeb and

Snail, respectively. I_{ext} represents an external signal that induces EMT by activating Snail. The function $P_y(\mu, n)$ represent the degradation due to the binding of miRNA with n binding sites (for details see Supplementary Information of [3]).

S2 - Parameter values

For a detailed discussion about the choice of the parameter values, please see the original references for Notch circuit [1, 2] and EMT circuit [3]. For most of the parameters, we kept the values used in the original references. A few parameter's values were adjusted in order to couple the circuit and are described below.

Table S1: *Standard parameters values of Notch-Delta-Jagged circuit used in the simulations. For more details please see [1, 2]. The symbol * indicates the values for 1 cell simulations for Figure 2 and ** for Figure 3.*

parameter	value	parameter	value	dimension
γ	0.1	γ_I	0.5	h^{-1}
g_N, g_D, g_J	8, 20, 70	g_D,g_J	(30,30)* (40, 15)**	mRNA per hour
k_T	$1e^{-5}$	k_C	$1e^{-4}$	h^{-1}
I_0	200	I_{0S}	300	# of molecules
n_N,n_D,n_J,n_S	2.0	n_F	1.0	
$\lambda_{I,N}, \lambda_{I,J}, \lambda_{I,D}, \lambda_{I,S}$	7.0, 2.0, 0.0, 6.5	λ_D^F, λ_J^F	3.0, 0.3	

The values of g_N, g_D and g_J which represents the production rate of the mRNA were chosen in order to keep the maximum number of proteins in the membrane up to approximately a few thousands per cell. This value is consistent with experimental results where the concentration of the proteins varies up to a few hundreds ng/ml [4]. Similarly, the number of NICD inside the nucleus varies up to a few hundreds and because of that, we select the threshold of the Hill function (I_0) to be 200 for the modulation of Notch, Delta and Jagged, and 300 for activation of Snail. We choose a lower Hill coefficiente for Jagged activation by NICD ($n_J = 2$ instead of $n_J = 5$ [1]) once the strong indirect activation of Jagged by NICD via miR200 is already considered in this framework. We assumed that Notch signaling dynamics should be considerably slower than EMT dynamics. Because of that we considered trans-activation and cis-inhibition to be 5 times slower than considered previously ($k_T = 1e^{-5}, k_C = 1e^{-4}$ instead of $k_T = 5e^{-5}, k_C = 5e^{-4}$ [1]). For all proteins, we considered a translation rate (k_P) of 100 proteins per mRNA per hour.

Table S2: *Standard parameters values of EMT circuit used in the simulations. We keept the same values as described in the original manuscript [3].*

parameter	value	parameter	value	dimension
$\gamma_{m_S}, \gamma_{m_Z}$	0.5	$\gamma_{\mu_{34}}, \gamma_{\mu_{200}}$	0.5	h^{-1}
$g_{\mu_{200}}$,$g_{\mu_{34}}$	2100, 1350	g_Z,g_S	11, 90	mRNA per hour
$n_{S,Z}, n_{S,\mu_{200}}, n_{\mu_{34}}$	2	$n_{Z,\mu_{200}}, n_{\mu_{200}}, n_{S,\mu_{34}}, n_{S,Z}$	3, 6, 1, 1	
$S^0_{m_Z}, S^0_{\mu_{200}}$	180K	μ^0_{34}, μ^0_{200}	10K	
$S^0_{\mu_{34}}, S^0_{m_S}$	300K,200K	$Z^0_{\mu_{34}}$	600K	
$\lambda_{Z,\mu_{200}}, \lambda_{S,\mu_{34}}, \lambda_{S,S}$	0.1	$\lambda_{Z,Z}, \lambda_{S,Z}, \lambda_{Z,\mu_{34}}$	7.5, 10.0, 0.2	

S3 - Model deduction

In this section we describe in details how the Notch and EMT circuit are integrated.

EMT module

The EMT framework is based on a computational model for microRNA-based chimeric (MBC) circuits, developed by our group. This framework explicitly models the various miR-mRNA com-

plexes formed by the binding/unbinding chemical reactions of miR and mRNA (for details see [3, 5]). Using this framework the equations that describe the dynamics of miR200 (μ_{200}), miR34 (μ_{34}), mZeb (m_Z), mSnail (m_S), Snail (S) and Zeb (Z) are:

$$\frac{d\mu_{200}}{dt} = g_{\mu_{200}} H^S(Z,\mu_{200}) H^S(S,\mu_{200}) - m_Z Y_\mu(\mu_{200},6) - m_J Y_\mu(\mu_{200},5) - \gamma_{\mu_{200}}\mu_{200} \tag{S9}$$

$$\frac{d\mu_{34}}{dt} = g_{\mu_{34}} H^S(S,\mu_{34}) H^S(Z,\mu_{34}) - m_S Y_\mu(\mu_{34},2) - m_N Y_\mu(\mu_{34},2) - m_D Y_\mu(\mu_{34},3) - \gamma_{\mu_{34}}\mu_{34} \tag{S10}$$

$$\frac{dm_Z}{dt} = g_Z H^S(Z,Z) H^S(S,Z) - m_Z Y_m(\mu_{200},6) - \gamma_{m_Z} m_Z \tag{S11}$$

$$\frac{dZ}{dt} = k_P m_Z L(\mu_{200},6) - \gamma_Z Z \tag{S12}$$

$$\frac{dm_S}{dt} = g_S H^S(S,S) H^S(I,S) - m_S Y_m(\mu_{34},2) - \gamma_{m_S} m_S \tag{S13}$$

$$\frac{dS}{dt} = k_P m_S L(\mu_{34},2) - \gamma_S S \tag{S14}$$

By assuming quasi-state approximation for the mRNA:

$$m_Z = \frac{g_Z H^S(Z,Z) H^S(S,Z)}{Y_m(\mu_{200},6) + \gamma_{m_Z}} \tag{S15}$$

$$m_S = \frac{g_S H^S(S,S) H^S(I,S)}{Y_m(\mu_{34},2) + \gamma_{m_S}} \tag{S16}$$

we have:

$$\begin{aligned}\frac{d\mu_{200}}{dt} =& g_{\mu_{200}} H^S(Z,\mu_{200}) H^S(S,\mu_{200}) - g_Z H^S(Z,Z) H^S(S,Z) P_y(\mu_{200},6) \\ & - g_J H^S(I,J) P_y(\mu_{200},5) - \gamma_{\mu_{200}}\mu_{200}\end{aligned} \tag{S17}$$

$$\begin{aligned}\frac{d\mu_{34}}{dt} =& g_{\mu_{34}} H^S(S,\mu_{34}) H^S(Z,\mu_{34}) - g_S H^S(S,S) H^S(I,S) P_y(\mu_{34},2) \\ & - g_D H^S(I,D) P_y(\mu_{34},3) - g_N H^S(I,N) P_y(\mu_{34},2) - \gamma_{\mu_{34}}\mu_{34}\end{aligned} \tag{S18}$$

$$\frac{dZ}{dt} = k_P g_Z H^S(Z,Z) H^S(S,Z) P_l(\mu_{200},6) - \gamma_Z Z \tag{S19}$$

$$\frac{dS}{dt} = k_P g_S H^S(S,S) H^S(I,S) P_l(\mu_{34},2) - \gamma_S S \tag{S20}$$

where $P_y(\mu,n) = Y_\mu(\mu,n)/(Y_m(\mu,n)+k_m)$ and $P_l(\mu,n) = L(\mu,n)/(Y_m(\mu,n)+k_m)$.

The functions $L(\mu,n)$, $Y_m(\mu,n)$ and $Y_\mu(\mu,n)$ are defined as:

$$L(\mu,n) = \sum_{i=0}^{n} l_i C_i^n M_i^n(\mu) \tag{S21}$$

$$Y_m(\mu,n) = \sum_{i=0}^{n} \gamma_{mi} C_i^n M_i^n(\mu) \tag{S22}$$

$$Y_\mu(\mu,n) = \sum_{i=0}^{n} \gamma_{\mu i} C_i^n M_i^n(\mu) \tag{S23}$$

where $M_i^n(\mu) = \frac{(\mu/\mu_0)^i}{(1+\mu/\mu_0)^n}$ and $C_i^n = \frac{n!}{i!(n-i)!}$ is the number of i-combinations of n items. The values of l_i, γ_{mi} and $\gamma_{\mu i}$ are described in table S3.

Table S3: *The values of l_i, γ_{mi} and $\gamma_{\mu i}$ (see more details see Supplementary Information of [3]).*

parameter	value	dimension
l_i	[1.0, 0.6, 0.3, 0.1, 0.05, 0.05, 0.05]	
γ_{m_i}	[,0.04, 0.2, 1.0, 1.0, 1.0, 1.0]	h^{-1}
γ_{μ_i}	[, 0.005, 0.05, 0.5, 0.5, 0.5, 0.5]	h^{-1}

Notch circuit

Within the framework previously introduced by our group [1, 2], Notch receptor (N) can interact either with the ligands of the same cell - Delta or Jagged (D or J) - known as *cis*-interaction, or with the ligands of the neighboring cell (D_{ext} or J_{ext}) - known as *trans*-interaction. While *cis*-interaction causes the degradation of both the interacting proteins, the *trans*-interaction leads to the cleavage of Notch receptor and the release of the Notch Intracellular Domain (NICD; represented as I in the model). The signal NICD indirectly activates Notch and Jagged, and represses Delta, thereby creating an asymmetry between Notch-Delta and Notch-Jagged interactions (Figure 1). A second asymmetry between the ligands comes from the asymmetric binding affinity between Notch-Delta and Notch-Jagged which is modulated by the glycosyltransferase Fringe. By modifying the Notch receptor, Fringe creates a modified Notch that is more likely to interact with Delta and less likely to interact with Jagged.

The deterministic equations for the dynamics of the amount of Notch (N), Delta (D), Jagged (J) in the membrane, their respective mRNA (m_N, m_D, m_J) and NICD (I) are given by:

$$\frac{dm_N}{dt} = g_N H^S(I,N) - m_N Y_m(\mu_{34},2) - \gamma_{m_N} m_N \tag{S24}$$

$$\frac{dN}{dt} = k_P m_N L(\mu_{34},2) - N[(k_{cD}D + k_{cJ}J) + (k_{tD}D_{ext} + k_{tJ}J_{ext})] - \gamma N \tag{S25}$$

$$\frac{dm_D}{dt} = g_D H^S(I,D) - m_D Y_m(\mu_{34},3) - \gamma_{m_D} m_D \tag{S26}$$

$$\frac{dD}{dt} = k_P m_D L(\mu_{34},3) - D(k_{cD}N + k_{tD}N_{ext}) - \gamma D \tag{S27}$$

$$\frac{dm_J}{dt} = g_J H^S(I,J) - m_J Y_m(\mu_{200},5) - \gamma_{m_J} m_J \tag{S28}$$

$$\frac{dJ}{dt} = k_P m_J L(\mu_{200},5) - J(k_{cJ}N + k_{tJ}N_{ext}) - \gamma J \tag{S29}$$

$$\frac{dI}{dt} = N(k_{tD}D_{ext} + k_{tJ}J_{ext}) - \gamma_I I \tag{S30}$$

where γ_{m_N}, γ_{m_D} and γ_{m_J} represents the degradation rate of the mRNA of Notch, Delta and Jagged, respectively and k_P represents the translation rate. The function $Y_m(\mu, n)$ represents the effect of n binding sites of miRNA on the degradation of the mRNA.

Once the time required for the proteins being exported to the membrane is much higher than the translation time, we assume the quasi-state approximation for the mRNAs. Then, the amount of mRNA for each protein are:

$$m_N = \frac{g_N H^S(I,N)}{Y_m(\mu_{34},2) + \gamma_{m_N}} \tag{S31}$$

$$m_D = \frac{g_D H^S(I,D)}{Y_m(\mu_{34},3) + \gamma_{m_D}} \tag{S32}$$

$$m_J = \frac{g_J H^S(I,J)}{Y_m(\mu_{200},5) + \gamma_{m_J}} \tag{S33}$$

Then, the equations for N, D, J and I are:

$$\frac{dN}{dt} = k_P g_N H^S(I,N) P_l(\mu_{34}, 2) - N[(k_{cD}D + k_{cJ}J) + (k_{tD}D_{ext} + k_{tJ}J_{ext})] - \gamma_N N \quad (S34)$$

$$\frac{dD}{dt} = k_P g_D H^S(I,D) P_l(\mu_{34}, 3) - D(k_{cD}N + k_{tD}N_{ext}) - \gamma_D D \quad (S35)$$

$$\frac{dJ}{dt} = k_P g_J H^S(I,J) P_l(\mu_{200}, 5) - J(k_{cJ}N + k_{tJ}N_{ext}) - \gamma_J J \quad (S36)$$

$$\frac{dI}{dt} = N(k_{tD}D_{ext} + k_{tJ}J_{ext}) - \gamma_I I \quad (S37)$$

For the case of many interacting cells, the variables N_{ext}, D_{ext} and J_{ext} should be replaced by N, D, J of the neighboring cells. For more details please see [1, 2].

S3 - Supplementary Figures

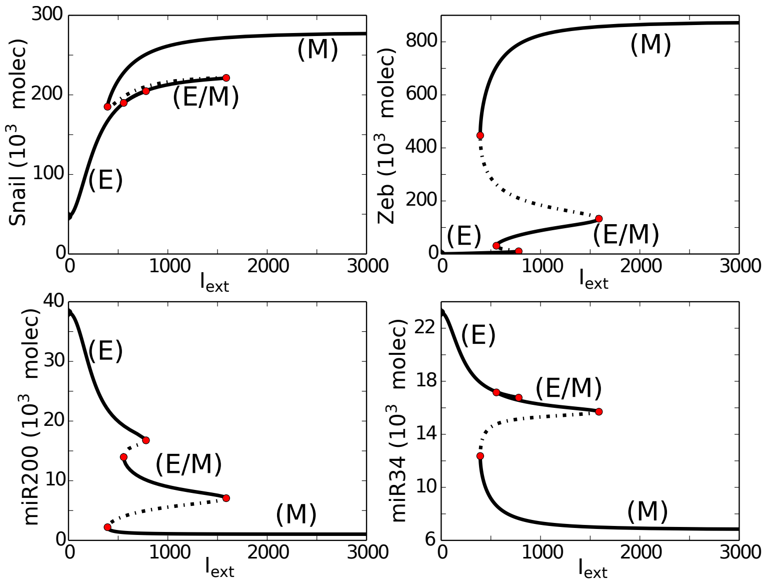

Figure S1: *Bifurcation curves of the levels of Snail, Zeb, miR34 and miR200 as a function of EMT-inducer (I_{ext}) signal that activates Snail.*

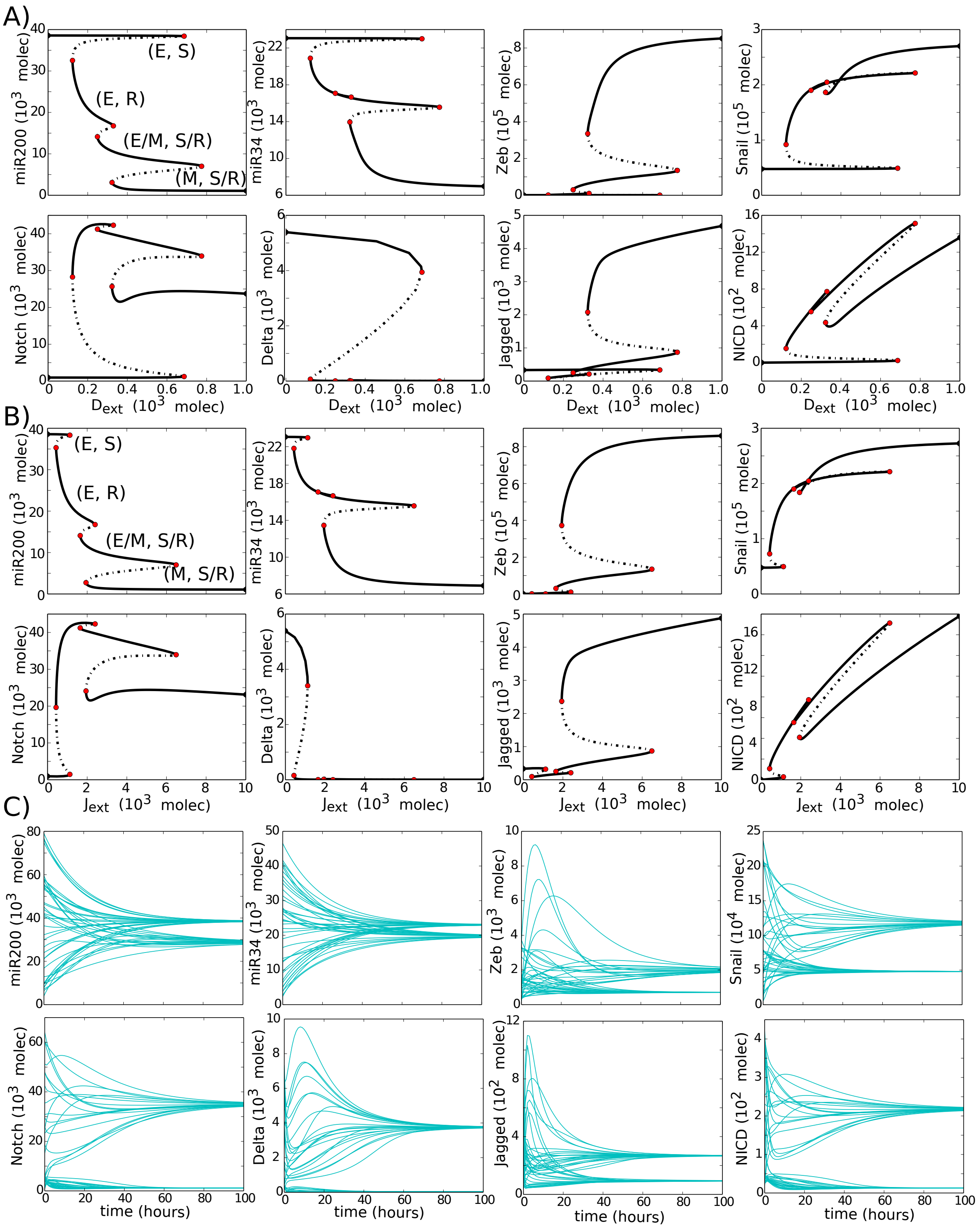

Figure S2: *Bifurcation curves of the levels of miR200, miR34, Zeb, Snail, Notch, Delta, Jagged and NICD as a function of A) amount of Delta in the neighboring cells (D_{ext}) and B) amount of Jagged in the neighboring cells (J_{ext}). C) Time dynamics of 40 simulations starting from random initial conditions for the same set of parameters of Fig. 3B.*

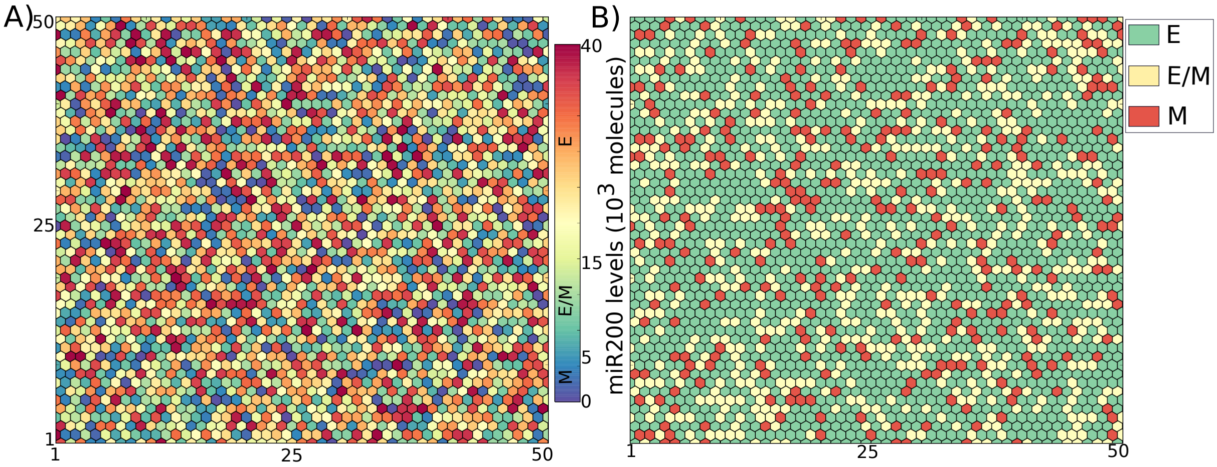

Figure S3: *Initial condition for the simulations presented in Figures 4, S4 and S7. The levels of each protein is chosen randomly from a uniform distribution where the range is defined based on the maximum and minimum values presented in Figure S3. A) Color represents the levels of miR200. B) Color represents the cell state: (E: $miR200 > 15000$, E/M: $15000 > miR200 > 5000$, M: $miR200 < 5000$ molecules)*

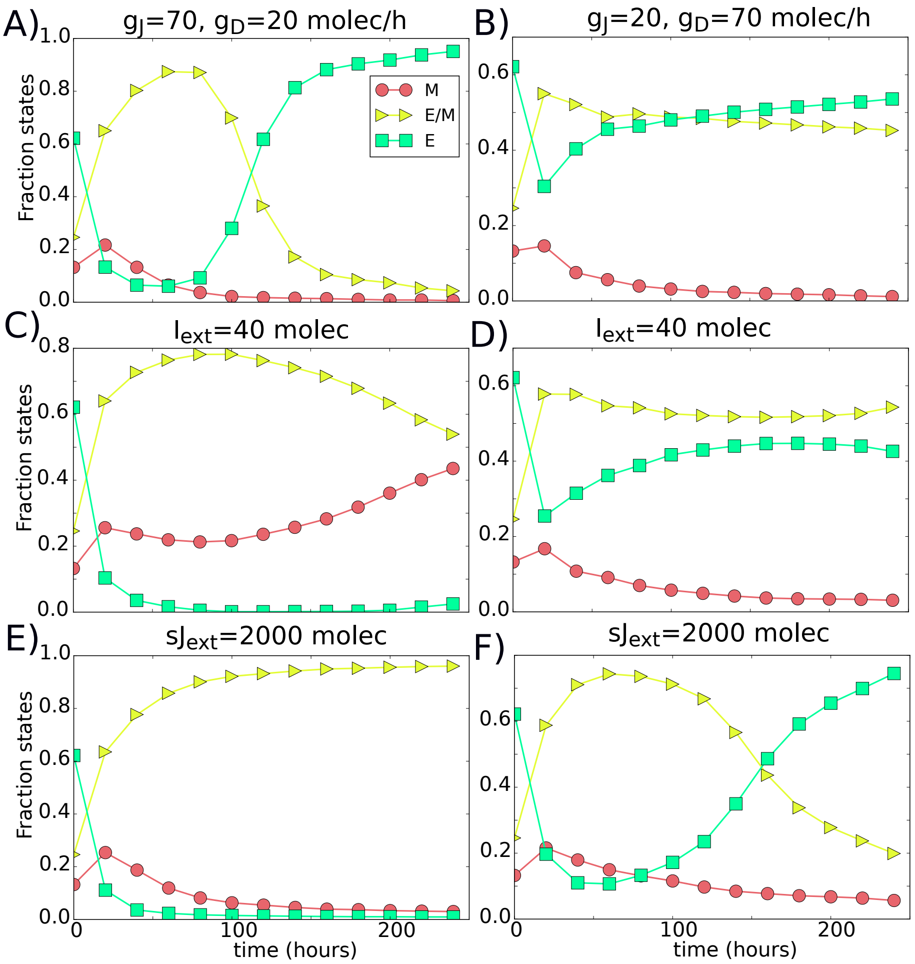

Figure S4: *Time evolution starting from the initial condition shown in Figure S3. Left column ($g_J > g_D$) and right column ($g_D > g_J$). A) No external signal: the clusters are transient. B) The cells keep the salt and pepper patterning between E and E/M even in the absence of any external signal. C) EMT-inducer signal stabilizes the clusters and drive the E/M cells to complete EMT. D) EMT-inducer signal stabilizes salt and pepper of E and E/M. E) External soluble Jagged stabilizes the clusters of E/M and increase them in time. F) External soluble Jagged disrupt salt-and-pepper and leads the E/M cells to start organizing in clusters.*

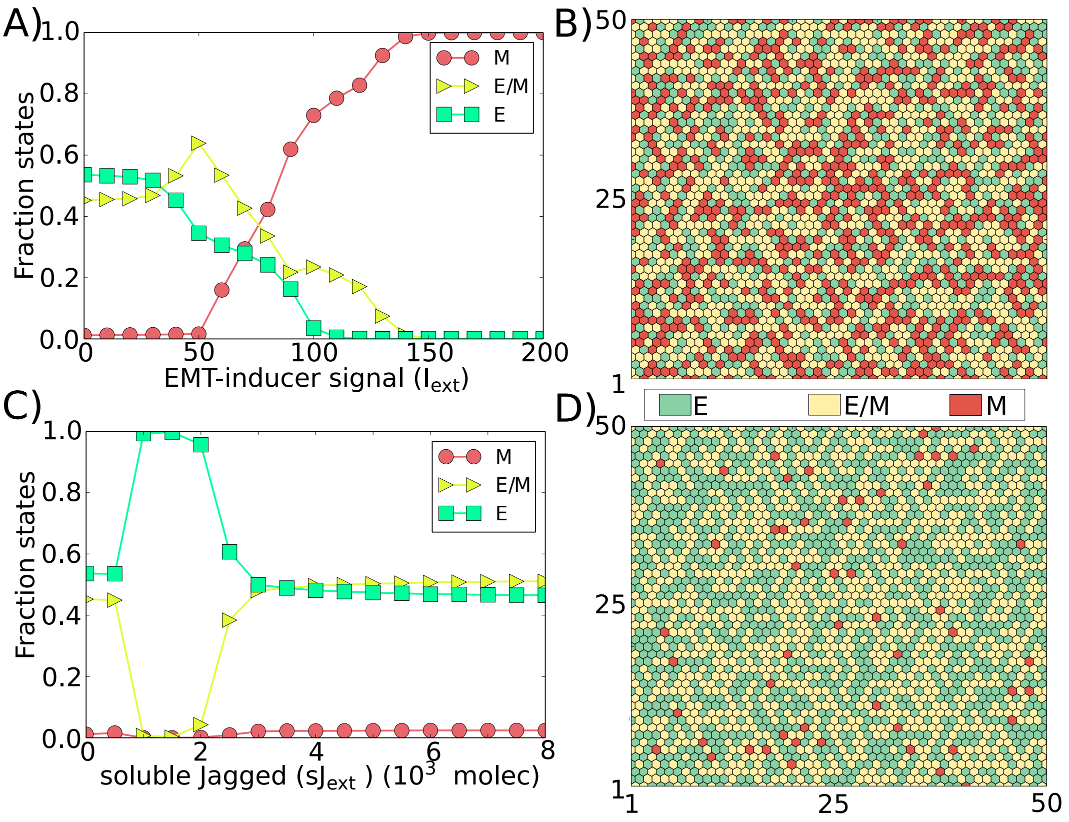

Figure S5: *Effect of external inducers of the Notch-EMT coupled circuit on tissue patterning. Simulation of a 2D layer of* 50*x*50 *cells interacting via Notch-Delta-Jagged signaling. A) Fraction of cells for each phenotype: epithelial (E), epithelial/mesenchymal (E/M) and mesenchymal (M) for different levels of an EMT-inducer signal* (I_{ext})*. B) Snapshot of the simulated tissue representing the spatial distribution of E, E/M and M cells for* $I_{ext} = 70$ *molecules,* $g_D = 70$ *and* $g_J = 20$ *molecules/h. A majority of the cells that undergo EMT adopt the M state. C) Fraction of cells for each phenotype for different levels of external soluble Jagged* (sJ_{ext}) *for* $I_{ext} = 0$ *molecules,* $g_D = 20$ *and* $g_J = 70$ *molecules/h. D) Snapshot of the simulated tissue representing the spatial distribution of E, E/M and M cells* $sJ_{ext} = 4000$ *molecules. The levels were measured after an equilibrium time of* 120*h, starting from the configuration presented in Figure 4C.*

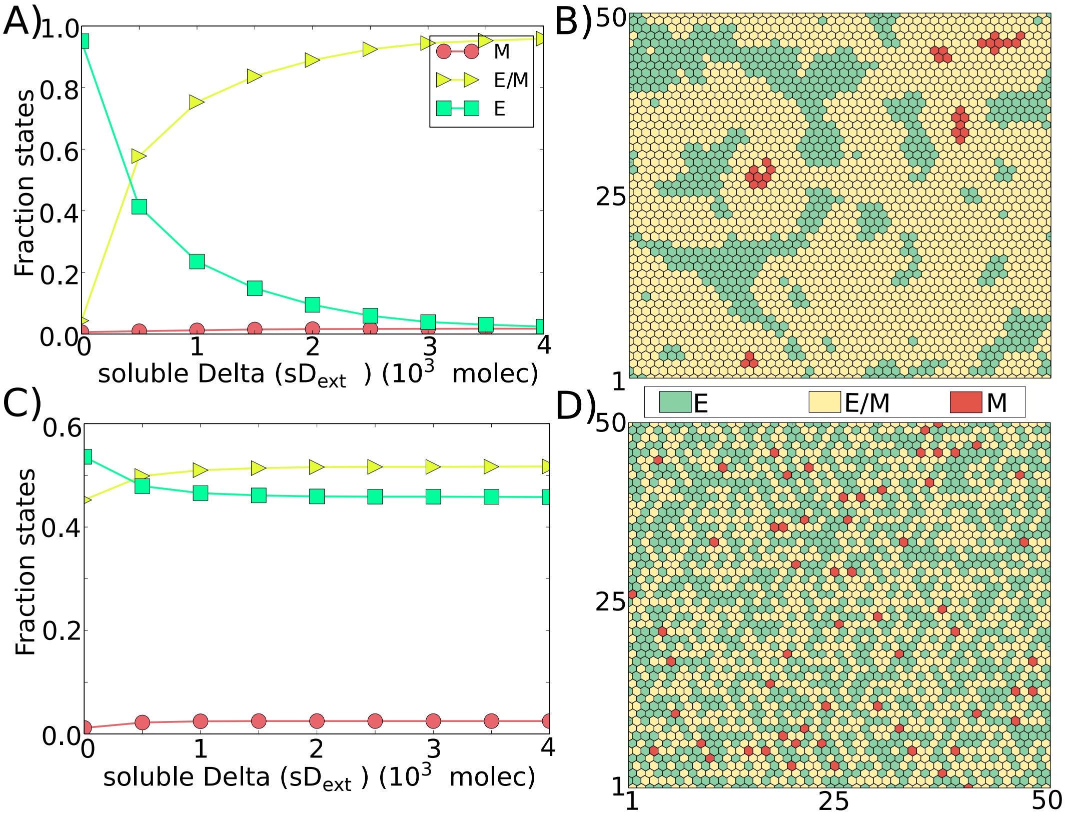

Figure S6: *Effect of soluble Delta on tissue patterning. Simulation of a 2D layer of* 50x50 *cells interacting via Notch-Delta-Jagged signaling. A) Fraction of cells for each phenotype: epithelial (E), epithelial/mesenchymal (E/M) and mesenchymal (M) for different levels of soluble Delta* (sD_{ext})*, for* $g_D = 20$ *and* $g_J = 70$ *molec/h. B) Snapshot of the simulated tissue representing the spatial distribution of E, E/M and M cells for* $D_{ext} = 1000$ *molecules,* $g_D = 20$ *and* $g_J = 70$ *molecules/h. C) same as A) for* $g_D = 70$ *and* $g_J = 20$ *molecules/h. D) Snapshot of the simulated tissue representing the spatial distribution of E, E/M and M cells for* $sD_{ext} = 1000$ *molecules,* $g_D = 70$ *and* $g_J = 20$ *molecules/h.*

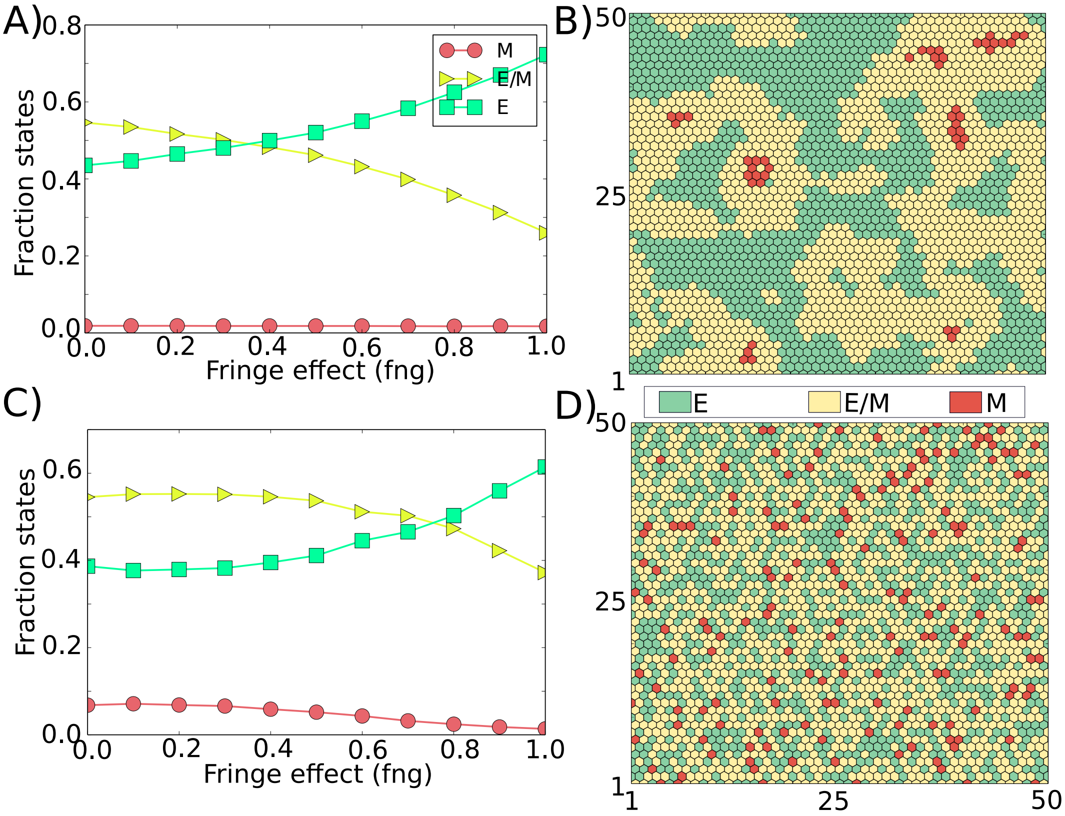

Figure S7: *Effect of Fringe on tissue patterning. Simulation of a 2D layer of* $50x50$ *cells interacting via Notch-Delta-Jagged signaling, starting from the initial condition described in Figure S4 after 120h. A) Fraction of cells for each phenotype: epithelial (E), epithelial/mesenchymal (E/M) and mesenchymal (M) for different values of Fringe effect (*fng*), for* $g_D = 20$ *and* $g_J = 70$ *molec/h. B) Snapshot of the simulated tissue representing the spatial distribution of E, E/M and M cells for the case of no Fringe effect (*$fng = 0.0$*, i.e,* $\lambda_D^F = \lambda_J^F = 1.0$*),* $g_D = 20$ *and* $g_J = 70$ *molec/h. Compare with figure 4D (intermediate Fringe effect). C) Same as A) for* $g_D = 70$ *and* $g_J = 20$ *molec/h. D) Snapshot of the simulated tissue representing the spatial distribution of E, E/M and M cells for the case of no Fringe effect (*$fng = 0.0$*, i.e,* $\lambda_D^F = \lambda_J^F = 1.0$*),* $g_D = 70$ *and* $g_J = 20$ *molec/h. Compare with figure 4C (intermediate Fringe effect). As* fng *increases, the values of* λ_D^F *and* λ_J^F *linearly increase and decrease respectively, such that at* $fng = 1.0$*,* $\lambda_D^F = 5.0$ *and* $\lambda_J^F = 0.1$*, i.e. Notch has higher binding affinity to Delta and lower to Jagged.*

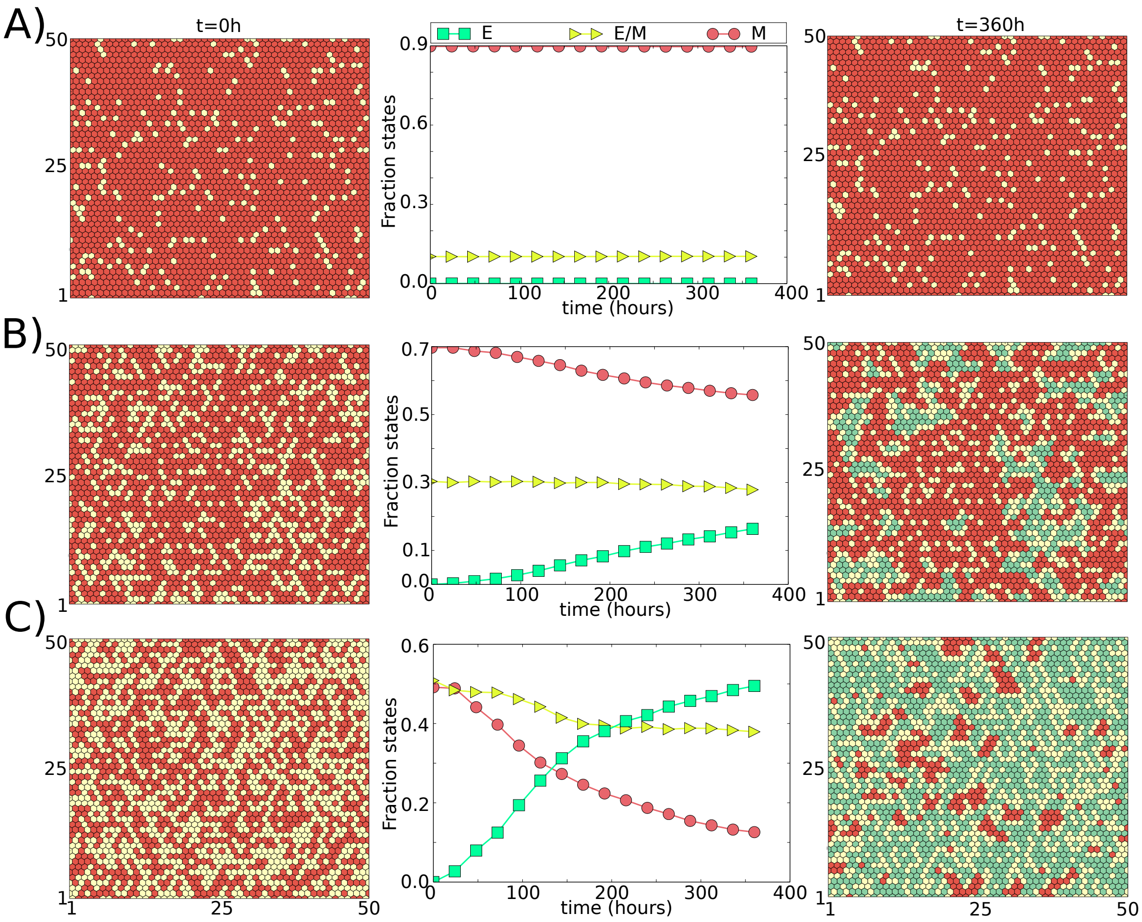

Figure S8: *Simulation of 50x50 cells interacting via N-D-J signaling for* $g_D = 70$ *and* $g_J = 20$ *molec/h. (middle) Fraction of cells adopting Epithelial (E), Epithelial/Mesenchymal (E/M) and Mesenchymal (M) phenotypes at different time points for the given initial condition. (left, right) Levels of miR200 for 50x50 hexagonal lattice at t=0 and t=360 hr. Red cells are in an M phenotype, yellow ones in a hybrid E/M phenotype and green ones in the E phenotype. A) Starting from 90% of cells in the M state and 10% in the E/M state. B) Starting from 70% of cells in the M state and 30% in the E/M state. C) Starting from 50% of cells in the M state and 50% in the E/M state.*

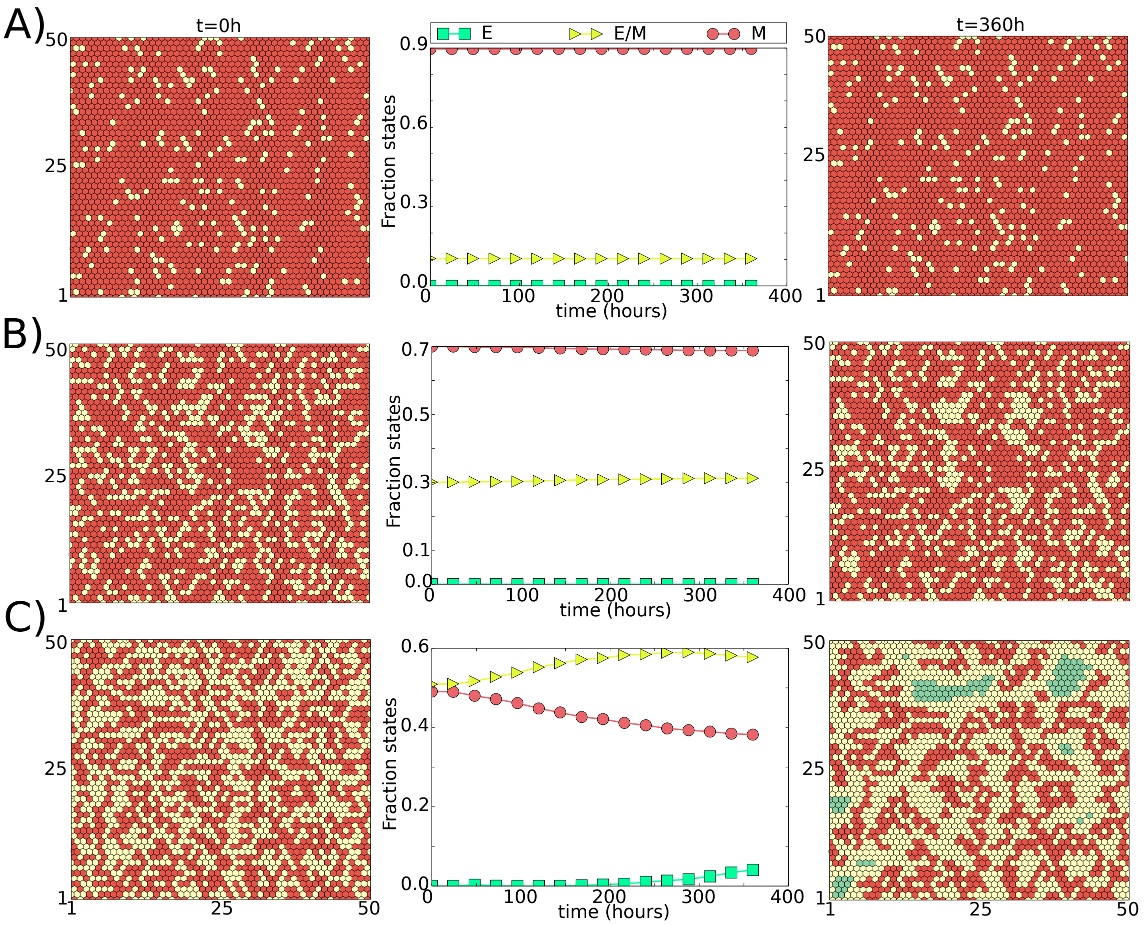

Figure S9: *Same as Figure S8 for* $g_D = 20$ *and* $g_J = 70$ *molec/h.*

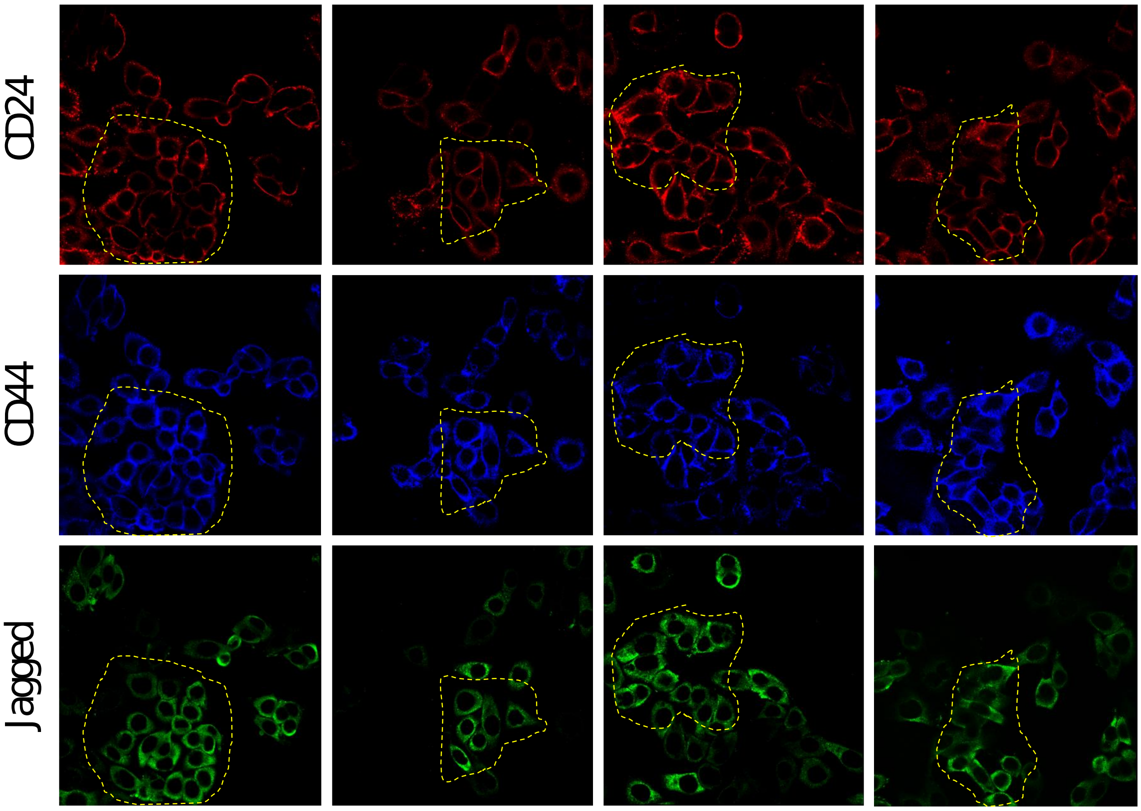

Figure S10: *Representative confocal microscopy shows MDA-MB-468 cells, which are predominantly of a CD44HiCD24Hi phenotype, for expression of Jagged. Note clustering of the Jagged expression with the E/M state.*

References

[1] Boareto, M., Jolly, M. K., Lu, M., Onuchic, J. N., Clementi, C., and Ben-Jacob, E. (2015). *JaggedDelta asymmetry in Notch signaling can give rise to a Sender/Receiver hybrid phenotype.* Proceedings of the National Academy of Sciences, 112(5), E402-E409.

[2] Jolly, M. K., Boareto, M., Lu, M., JoseN, O., Clementi, C., and Ben-Jacob, E. (2015). *Operating principles of NotchDeltaJagged module of cellcell communication.* New Journal of Physics, 17(5), 055021.

[3] Lu, M., Jolly, M. K., Levine, H., Onuchic, J. N., and Ben-Jacob, E. (2013). *MicroRNA-based regulation of epithelial–hybrid–mesenchymal fate determination* Proceedings of the National Academy of Sciences, 110(45), 18144-18149.

[4] Amsen, D. et al. (2004). *Instruction of distinct CD4 T helper cell fates by different notch ligands on antigen-presenting cells.* Cell, 117(4), 515-526.

[5] Lu, M., Jolly, M. K., Gomoto, R., Huang, B., Onuchic, J., and Ben-Jacob, E. (2013). *Tristability in cancer-associated microRNA-TF chimera toggle switch.* The Journal of Physical Chemistry B, 117(42), 13164-13174.
